# A non-linear detection of phospho-histone H2AX in EA.hy926 endothelial cells following low-dose X-irradiation is modulated by reactive oxygen species

**DOI:** 10.1186/1748-717X-9-80

**Published:** 2014-03-22

**Authors:** Martin Large, Sebastian Reichert, Stephanie Hehlgans, Claudia Fournier, Claus Rödel, Franz Rödel

**Affiliations:** 1 Department of Radiotherapy and Oncology, Goethe-University of Frankfurt, Theodor-Stern-Kai 7, 60590 Frankfurt am Main, Germany; 2 Department of Biophysics, GSI Helmholtz Center for Heavy Ion Research, Planckstraße 1, 64291 Darmstadt, Germany

**Keywords:** Low-dose X-irradiation, γH2AX, ROS, SOD, Endothelial cells

## Abstract

**Background:**

A discontinuous dose response relationship is a major characteristic of the anti-inflammatory effects of low-dose X-irradiation therapy. Although recent data indicate an involvement of a variety of molecular mechanisms in these characteristics, the impact of reactive oxygen species (ROS) production to give rise or contribute to these phenomena in endothelial cells (EC) remains elusive.

**Material and methods:**

HUVEC derived immortalized EA.hy926 cells were stimulated by tumor necrosis factor-α (TNF-α, 20 ng/ml) 4 h before irradiation with doses ranging from 0.3 to 1 Gy. To analyse DNA repair capacity, phospho-histone H2AX foci were assayed at 1 h, 4 h and 24 h after irradiation. ROS production and superoxide dismutase (SOD) activity were analysed by fluorometric 2′,7′-dichlorodihydrofluorescein-diacetate (H2DCFDA) and colorimetric assays. A functional impact of ROS on γH2AX production was analysed by treatment with the scavenger N-acetyl-L-cysteine (NAC).

**Results:**

Irrespective of stimulation by TNF-α, EA.hy926 cells revealed a linear dose response characteristic of γH2AX foci detection at 1 h and 4 h after irradiation. By contrast, we observed a discontinuity in residual γH2AX foci detection at 24 h after irradiation with locally elevated values following a 0.5 Gy exposure that was abolished by inhibition of ROS by NAC. Moreover, SOD protein expression was significantly decreased at doses of 0.5 Gy and 0.7 Gy concomitant with a reduced SOD activity.

**Conclusion:**

These data implicate a non-linear regulation of ROS production and SOD activity in EA.hy926 EC following irradiation with doses < 1 Gy that may contribute to a discontinuous dose-response relationship of residual γH2AX foci detection.

## Background

For decades an anti-inflammatory and analgetic effect of low-dose X-irradiation (LD-RT) has been well established in the treatment of a plethora of benign diseases and chronic degenerative disorders [1,2] with empirically identified single doses < 1 Gy to be most effective in the clinical setting [3-5]. Although the knowledge of the underlying cellular and molecular mechanisms is still at an early stage, a modulation of endothelial cell (EC) activity has already been proven to comprise a key element in the therapeutic effects of LD-RT [6]. For instance, a hampered adhesion of peripheral blood mononuclear (monocytes, lymphocytes) and polymorphonuclear leukocytes (granulocytes) to EC was shown to result from an elevated secretion of the anti-inflammatory cytokine transforming growth factor β1 (TGF-β1), elevated levels of X-chromosome linked inhibitor of apoptosis protein (XIAP) and transcription factor nuclear factor-κB (NF-κB) DNA-binding and transcriptional activity [7-10]. Moreover, a hampered adhesion and consequently reduced immune cell infiltration [11] is further supported by a lowered expression of the adhesion molecule E-selectin with a local minimum following 0.3-0.5 Gy exposure [9,12]. Strikingly, the mechanisms explored so far display comparable non-linear dose effect relationships, a common hallmark of bystander and non (DNA)-targeted effects of low-dose irradiation [13] and the phenomenon of low-dose hypersensitivity reported for cellular survival [14]. These mechanisms are supposed to originate from an overlap of multiple molecular processes that may be initiated at various dose thresholds [15].

Reactive oxygen species (ROS) like superoxide (O_2_^•-^), Hydroxyl (OH^•^) and Hydroperoxyl (HO_2_^•^) ions formed as natural by-product of the mitochondrial electron transport chain and by NADPH oxidase activity display important roles in the regulation of cell signalling, cellular homeostasis, cell death and mutagenesis of DNA [16,17]. These regulatory effects include reversible oxidation of serine/threonine phosphatases and kinases, e.g. mitogen-activated protein kinase (MAPK), metalloproteases and activation of transcription factors like NF-κB and activating protein 1 (AP1) [18]. Moreover, following environmental stress, including ionising radiation and heat exposure, ROS levels increase dramatically resulting in significant damage to cellular structures and induction of DNA double-strand breaks (DSBs) [19].

A putative interrelationship between DNA damage repair and a discontinuous dose response relationship following low-dose irradiation was recently suggested [20]. By assessing serine 139 phosphorylated histone γH2AX foci induction, a marker of radiation-induced DSBs [21], a biphasic behaviour of γH2AX foci induction with a low-dose hypersensitivity in whole blood and less pronounced for isolated T-lymphocytes after X-irradiation was reported in line with a delayed repair with 40% of initial γH2AX foci persisting 24 hours post-irradiation [20]. A mechanistic involvement of ROS in the modulation of these non-linear dose response effects, however, remains to be established. Thus, in the present study we analysed radiation effects with a particular focus on low-dose (0.3 -1 Gy) irradiation of EA.hy926 EC with respect to γH2AX foci induction, ROS production and SOD activity.

## Material and methods

### Cell culture and stimulation of endothelial cells

The human endothelial cell (EC) line EA.hy926 was established by fusion of human umbilical vein endothelial cells (HUVEC) and the adenocarcinoma epithelial cell line A549 [22]. EA.hy926 cells were grown in Dulbecco’s modified Eagle’s medium (DMEM; Invitrogen, Karlsruhe, Germany) supplemented with 10% foetal calf serum (FCS; PAA, Cölbe, Germany) and 50 U/ml penicillin and 50 μg/ml (streptomycin) (Sigma-Aldrich, Munich, Germany). Primary HUVEC were isolated from umbilical vein vascular wall according to a technique described in [23], plated on fibronectin-coated plates and cultured in DMEM supplemented with 5% endothelial cell growth supplement (ECGS) (Invitrogen, Karlsruhe, Germany) and 1% penicillin/streptomycin. Cell culture was performed at 37°C in a 5% CO_2_ incubator with 95% humidity. For inflammatory stimulation, cells were treated according to pilot experiments with the cytokine TNF-α (Miltenyi Biotec, Bergisch-Gladbach, Germany) at a concentration of 20 ng/ml at 4 h before irradiation.

### Treatment with ROS scavenger and irradiation procedure

ROS scavenger N-acetyl-L-cysteine (NAC) was applied at a concentration of 10 mM 4 h before irradiation and maintained in the cultures during repair incubation (24 h). For irradiation purposes, EA.hy926 were exposed to single doses of 0.3 to 1 Gy photons using a linear accelerator (SL75/5, Elekta, Crawley, UK) with 6 MeV/100 cm focus-surface distance and a dose rate of 4 Gy/min. Mock-treated controls were kept in parallel at ambient temperature in the accelerator control room.

### Immunofluorescence quantification of phospho-histone H2AX foci formation

EA.hy926 EC were grown on glass coverslips in 6-well plates for 48 h, treated with TNF-α, NAC or were mock-treated and irradiated as described before. At 1 h, 4 h and 24 h post irradiation cells were fixed with 3.7% paraformaldehyde (15 min, AppliChem, Darmstadt, Germany) at room temperature (RT), and permeabilization was performed by addition of 0.25% Triton-X 100 in PBS for 15 min, followed by blocking in 3% bovine serum albumin (BSA) in PBS for 60 min. Next, EA.hy926 cells were incubated with anti-phospho-histone H2AX specific (γH2AX, 1:1000, Millipore, Darmstadt, Germany) and anti-centromere protein F (CENP-F) primary antibodies (1:2000, Santa Cruz, Heidelberg, Germany) to discriminate cells in G1 and S/G2 cell cycle phases [24] followed by appropriate Alexa-labelled secondary antibodies (Invitrogen, Darmstadt, Germany). Subsequently, nuclei were counterstained with DAPI solution (Invitrogen) and coverslips were mounted with Vectashield (Vector Laboratories, Peterborough, UK). Images were taken using an AxioImager Z1 microscope, equipped with an Axiocam camera and Axiovision 4.6 software (Zeiss, Göttingen, Germany). For quantification of γH2AX foci formation 40 G1 and 40 S/G2 phase cells as differentiated by CENP-F signal intensity were evaluated per experiment. At least three independent experiments were performed for each data point.

### Measurement of ROS levels and SOD activity assay

Intracellular ROS levels were determined by flow cytometry using the cell membrane permeable dye 2′,7′-dichlorodihydrofluoresceindiacetate (H_2_DCFDA: DCF assay) as described in [25]. Prior to harvesting, cells were incubated for 90 minutes with the dye at a concentration of 2 μM in serum-free medium. At indicated times cells were trypsinized on ice and analyses were performed using a FACSCalibur® cytometer and Cellquest Pro software (Becton Dickinson, Heidelberg, Germany). The mean fluorescence of mock-treated cells was subtracted to eliminate unspecific background intensity for every sample. To assess SOD activity a colorimetric activity kit (Sigma-Aldrich) was used according to the manufacturer’s instructions. Briefly, 3 × 10^3^ cells per well were plated in 96-well plates 24 h before irradiation. At indicated time points (up to 24 h) medium was removed and cells were incubated with 200 μl of working solution buffer (WST, Sigma-Aldrich) and 20 μl of enzyme working solution for 20 min. Absorbance was determined spectrophotometrically at a wavelength of 450 nm using an ELISA reader (Victor Wallac multilabel-reader, Perkin-Elmer, Waltham, USA).

### Immunoblotting

For Western immunoblotting, EA.hy926 cells were lysed in radioimmunoprecipitation assay buffer (RIPA) as described in [26]. Equal amounts of protein (20 μg) as determined by a bicinchoninic acid (BCA) protein assay (Pierce, Rockford, USA) were separated on 10% SDS polyacrylamide gels, transferred to nitrocellulose membranes (GE Healthcare, Munich, Germany), probed with anti-SOD-1 antibodies (1:2000, Cell Signaling, Frankfurt am Main, Germany) or anti-β-actin antibodies (1:10000, Sigma-Aldrich) diluted in 5% non-fat dry milk in Tris/Borat/Tween (TBS-T) buffer and appropriate horseradish-peroxidase (HRP)-conjugated secondary antibodies (Santa Cruz, Heidelberg, Germany). Blots were subsequently developed by an enhanced chemoluminescence detection system (Pierce ECL, Thermo Scientific, Hudson, USA) and autoradiography (Amersham Hyperfilm ECL, GE Healthcare). Densitometric analysis was performed using ImageJ software (US National Institutes of Health, Bethesda, USA).

### Statistical analysis

Experimental data are presented as mean ± standard deviations from at least three or more independent experiments. To test statistical significance, a two-sided unpaired Student’s t-test was performed using Excel® software (Microsoft, Unterschleißheim, Germany). Results were considered statistically significant if a p-value of less than 0.05 was reached.

## Results

### Phospho-histone H2AX foci detection in EA.hy926 EC following low-dose irradiation

**Figure 1:**
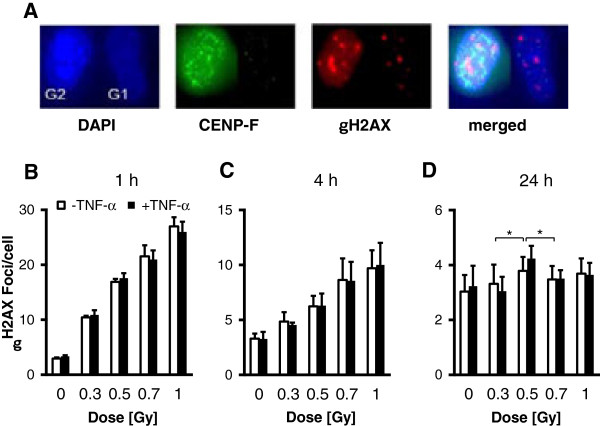
**Dose and time kinetics of γH2AX foci detection in EA.hy926 EC following low-dose X-irradiation.** EA.hy926 EC were plated onto coverslips and grown to confluence. At 4 h before irradiation with the doses indicated, ECs were stimulated with TNF-α (20 ng/ml), while mock-treated cells served as a control. At 1 h **(B)**, 4 h **(C)** and 24 h **(D)** post irradiation, cells were fixed, stained for γH2AX, DAPI and CENP-F **(A)** and data of a total of 80 nuclei (40 G1 and 40 S/G2) were combined for a single data point. Data represent means ± SD from at least three independent experiments. Asterisks indicate significant differences (p < 0.05) vs. 0.3 Gy and 0.7 Gy irradiated ECs.

EA.hy926 EC were plated onto glass coverslips, grown to 95% confluence and were stimulated with TNF-α (20 ng/ml) or mock treated 4 h before irradiation with single doses of 0.3, 0.5, 0.7 or 1 Gy. Induction of DNA DSBs was investigated by quantifying γH2AX foci formation at 1 h and 4 h and by counting residual foci at 24 h after irradiation. Phospho-histone H2AX signals may differ in a cell-cycle dependent manner with a doubled amount of DNA in the G2 phase [24]. Thus, to improve accuracy of γH2AX foci measurement, both cells in the G1 and G2 phase as differentiated by the intensity of the centromere protein F (CENP-F) signal (Figure 1A) were taken into consideration. At early time points (1 h, 4 h) we observed, irrespective of stimulation with TNF-α, a linear dose response relationship of γH2AX foci induction (Figure 1B, C). By contrast, at 24 h after irradiation the number of residual γH2AX foci was significantly (p < 0.05) elevated after a 0.5 Gy exposure and TNF-α stimulation as compared to irradiation with doses of 0.3 Gy and 0.7 Gy (Figure 1D). As depicted in Additional file 1: Figure S1, these characteristics could be observed in both G1 and S/G2 cells (with expected elevated numbers of γH2AX foci) indicating that the discontinuity in γH2AX detection at 24 h after low-dose irradiation is not related to cell cycle distribution.

### ROS expression and SOD activity in EA.hy926 EC following low-dose X-irradiation

**Figure 2:**
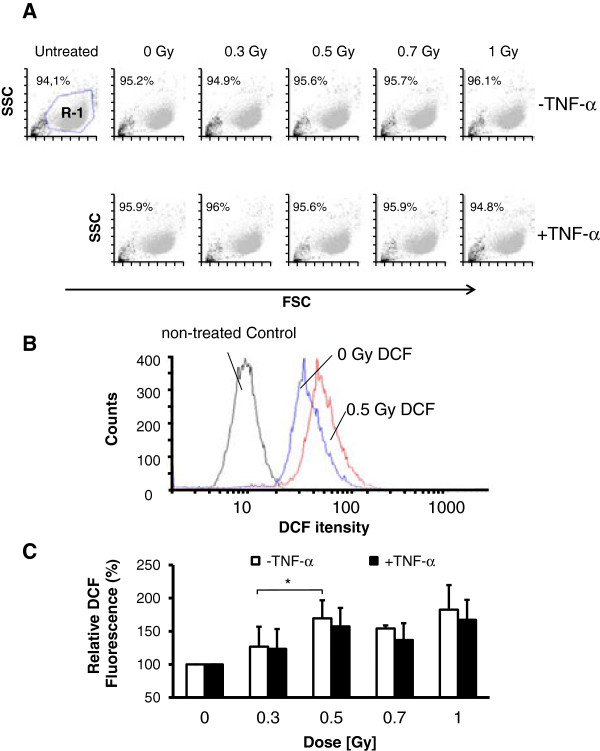
**ROS levels in EA.hy926 ECs following low-dose X-irradiation. (A)** Representative flow cytometer dot plots forward scatter (FSC) vs side scatter (SSC) for each treatment regimen. R-1 indicates the population of cells used for the analysis of 2′,7′-dichlorodihydrofluoresceindiacetate (DCF) fluorescence. **(B)** Representative histograms showing an increase of DCF fluorescence as a marker of ROS production in TNF-α stimulated, non-irradiated (blue line) and irradiated (0.5 Gy, red line) cells. Non-DCF treated cells (black line) served as a control and were subtracted in every quantification. **(C)** Quantification of relative DCF-fluorescence in EA.hy926 ECs at 24 h after irradiation with the doses indicated. Data represent means ± SD (n = 4). *p < 0.05 vs. 0.3 Gy irradiated EC.

Induction of ROS is known to result in DSBs [27]. To investigate a relationship between ROS production and the non-linear detection of γH2AX foci, EA.hy926 EC were stimulated with TNF-α 4 h before irradiation or mock-treatment and ROS levels were analysed using a flow cytofluorometric DCF assay (Figure 2B). As a control forward/side scatter analyses were performed (Figure 2A), indicating that neither irradiation nor stimulation with TNF-α alters cellular morphology. As depicted in Figure 2C, a biphasic appearance of DCF fluorescence with locally elevated values following irradiation with 0.5 Gy became evident at 24 h irrespective of inflammatory stimulation of the EC by TNF-α.

**Figure 3:**
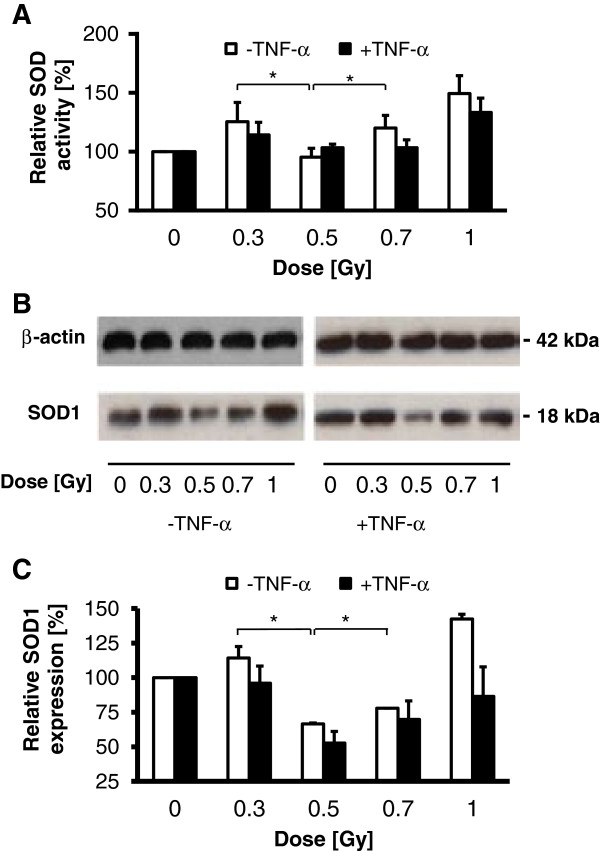
**SOD activity and SOD1 protein expression in EA.hy926 cells following low-dose X-irradiation. (A)** Relative SOD activity as analysed 24 h after irradiation by using a colorimetric activity assay. Data represent means ± SD (n = 3). *p < 0.05 vs. 0.3 Gy and 0.7 Gy treated cells. **(B)** Western immunoblots from total cellular proteins at 24 h after irradiation using antibodies against SOD1 and β-actin for loading control. Data are displayed as one representative out of three independent experiments. **(C)** Reduction of SOD1 protein expression normalized to β-actin control as determined by densitometric analysis using the ImageJ software package from two independent experiments including data from **(B)**. *p < 0.05 vs. 0.3 Gy and 0.7 Gy irradiated cells.

To further explore underlying molecular mechanisms implicated in the discontinuous induction of ROS following LD-RT, we next focused on the expression and enzymatic activity of SOD, reported to be involved in anti-oxidant defence by the conversion of superoxide (O_2_^-^) to H_2_O_2_ and O_2_[28]. As shown in Figure 3A and Figure 3B SOD activity and SOD-1 protein expression, respectively, displayed a discontinuous dose dependency with a relative minimum at 0.5 Gy as proven by colorimetric assay and Western immunoblotting. Employing densitometric analyses the relative amount of protein reduction was quantified. Exposure of EA.hy926 EC to 0.5 Gy and less pronounced to 0.7 Gy resulted in a 50% and 30% reduction of SOD-1 expression in stimulated ECs, as compared to mock-irradiated controls, respectively (Figure 3C). Comparable results were exemplarily obtained by using primary HUVEC cells (Additional file 2: Figure S2).

### A discontinuous γH2AX foci expression is abolished by treatment with the ROS scavenger NAC

**Figure 4:**
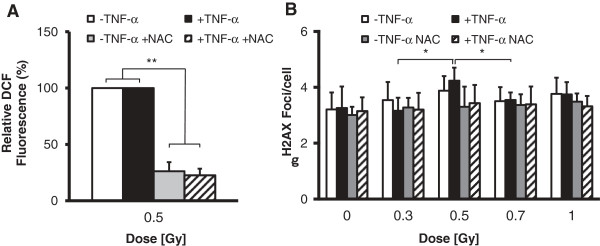
**NAC treatment abolishes the non-linear characteristics of γH2AX induction in EA.hy926 EC following low-dose X-irradiation.** EA.hy926 EC were stimulated with TNF-α (20 ng/ml) or mock-treated and were irradiated in the presence of NAC (10 mM, applied 4 h before irradiation). At 24 h after irradiation relative DCF-fluorescence **(A)** and γH2AX foci induction **(B)** were analysed as described before. Data represent means ± SD from at least three independent experiments. *p < 0.05 vs. 0.3 Gy and 0.7 Gy irradiated EC. **p < 0.01 vs. relative DCF controls.

Finally, to investigate an interrelationship between ROS induction and discontinuous γH2AX foci detection at 24 h after LD-RT, EA.hy926 EC were irradiated in the presence of the ROS scavenger NAC. In pilot experiments (Figure 4A) NAC treatment (10 mM) was proven to result in a significant reduction in total ROS levels to 20% of mock-treated controls. As illustrated in Figure 4B, the non-linear appearance of γH2AX foci was completely abolished upon pre-treatment with NAC, indicating a direct correlation between ROS production and the biphasic behaviour of γH2AX detection.

## Discussion

Although considerable progress has been achieved in the understanding of immune modulatory effects of ionising radiation, the underlying molecular mechanisms are presently not fully resolved. In line with that, high dose exposure with single doses exceeding 2 Gy displays a pronounced pro-inflammatory effect [29] whereas irradiation with single doses below 1 Gy experimentally and clinically reveal anti-inflammatory properties [2,4,6]. This may implicate the involvement of complex mechanisms of DNA damage response and immune modulation differentially operating at different dose levels. In that context, EC may comprise ideal targets for modulatory properties of low-dose and high-dose irradiation exposure due to their crucial role in the regulation of the local inflammatory process both by their ability to recruit leukocytes to the site of local inflammation and by expressing a variety of cytokines/chemokines essential for the inflammatory cascade [30]. Although recent data imply an involvement of a variety of molecular mechanisms in the anti-inflammatory characteristics of EC following low-dose irradiation [3], the impact of ROS production to give rise or contribute to these effects in EC remains elusive.

Here we show a linear dose response relationship for γH2AX foci induction at 1 h and 4 h after irradiation irrespective of stimulation of the cells in a pro-inflammatory manner by adding TNF-α. This may indicate that induction of DSBs and early DNA damage repair at low-dose irradiation, at least in EA.hy926 EC, may not be altered in an inflammatory surrounding characteristic for benign diseases treated clinically with LD-RT. Moreover, DNA damage repair response is considered to be linear with dose [31,32], which is in agreement with our data on the linearity of γH2AX induction at early times (1 h, 4 h) after irradiation. At later times (24 h), however, we observed a discontinuous dose-response relationship of residual γH2AX foci along with a non-linear detection of ROS with elevated levels following a 0.5 Gy exposure. This further confirms a close relationship between intracellular ROS and the induction of histone γH2AX foci as a marker of DNA damage [21]. In line with that, cellular ROS production is tightly regulated by coordinated activities of pro-oxidant and anti-oxidant defence mechanisms. To further elucidate mechanisms that may contribute to the non-linear induction of γH2AX foci, we thus analysed the activity of the detoxifying enzyme SOD that dismutates O_2_^•-^ into H_2_O_2_ with the latter to be degraded into H_2_O and O_2_ by catalase and glutathione peroxidase activity [33]. Data on the expression of SOD following low-dose irradiation, however, are controversial at present. Similar to our findings, they include a reduction in SOD activity in spleens of healthy BALB/C mice following total body irradiation with a dose of 0.4 Gy [34]. By contrast, they further comprise reports on increased mRNA expression following irradiation with a dose of 0.2 Gy or 0.5 Gy in splenic tissue of BALB/c or C57BL/6NJcl mice suffering from hepatopathy or cold brain injury [35,36]. These results pinpoint to a cell type and environment related regulation of anti-oxidative defence mechanisms that should be addressed in continuative investigations on the role of SOD in low-dose irradiation responses.

Notably, Kang et al. recently demonstrated that ROS induction after treatment of osteosarcoma and mammary epithelial cells with the radiation mimetic neocarzinostatin is, at least in part, mediated by γH2AX overexpression or DNA damage triggered γH2AX accumulation. Moreover, ROS induction by H2AX was abrogated by treatment with NAC, knockdown of the NADP(H) oxidase Nox1 and by a dominant negative Ras-related C3 botulinum toxin substrate 1 (Rac1) mutant (Rac1N17) indicating an involvement of the Nox1 and Rac1 GTPase pathway [37]. These findings thus point to a more complex and reciprocal regulation of γH2AX and ROS production that may further contribute to a discontinuous appearance of γH2AX foci in EA.hy926 ECs.

In this study we focused on the human endothelial cell line EA.hy926 which has been established by fusion of primary HUVEC with the adenocarcinoma epithelial cell line A549 [22]. As we can’t exclude that the cancerous fusion partner A549 may influence some properties of EA.hy926 cells as shown for apoptosis induction [38], we performed exemplary experiments on SOD expression and activity in primary HUVEC, showing a similar dose response relationship (Additional file 2: Figure S2). A comparability is further supported by studies indicating similarities between EA.hy926 ECs and HUVEC in terms of adhesion properties and surface marker expression if stimulated with TNF-α [39]. Thus, we consider that the EA.hy926 line may comprise a valuable system to investigate the role of SOD and DNA damage response following low-dose exposure.

A discontinuous regulation of ROS production following X-irradiation in a comparable dose range between 0.3 and 0.6 Gy is also reported in stimulated murine RAW 264.7 macrophages when they mount an oxidative burst [40]. However, as compared to elevated levels at a dose of 0.5 Gy in our investigation, a significant reduction of ROS production was observed in these macrophages. This may further indicate that the variety of regulatory effects observed after low-dose X-ray exposure may reflect different functional consequences that are specific for a given cell type (ECs vs. macrophages) or cellular environment.

Applying DNA binding and transcriptional activity assays, we recently reported on a biphasic activity of the transcription factor NF-κB in stimulated EA.hy926 ECs at 24 h after irradiation with locally elevated values following a 0.5 Gy exposure [8]. Moreover, NF-κB activation has been shown to be regulated by ROS (H_2_O_2_) by both the classical (canonical) and by alternative pathways including atypical inhibitor κBα (IκBα) phosphorylation independently of IκB kinase (IKK) [41]. Although experimentally not proven at present, it is tempting to speculate that elevated levels of ROS at a dose of 0.5 Gy may further contribute to an increased NF-κB activity and as a consequence to increased secretion of the cytokine TGF-β1 and the anti-adhesive efficacy of LD-RT [7,9]. This assumption is further supported by a very recent report indicating that ROS comprises a regulator of adhesion molecules very late antigen-4 (VLA-4) and vascular cell adhesion molecule-1 (VCAM-1) mediated monocyte/macrophage adhesion to EC following irradiation with a dose of 0.5 Gy [42].

In conclusion our data implicate a non-linear regulation of SOD activity and ROS production in EC following irradiation with doses < 1 Gy that may contribute to a discontinuous dose-response relationship of phospho-histone H2AX detection and a putative discontinuous behaviour of DNA damage response. A mechanistic involvement of DNA damage repair mechanisms in the modulation of these non-linear dose response effects remains to be established. However, one may assume that a discontinuous detection of residual γH2AX foci in our investigation is related to the phenomenon of low-dose hyper-radiosensitivity (HRS) and induced radioresistance (IRR), which have been reported for cellular survival at doses below 0.3 Gy and in the dose range of 0.3 Gy to 0.6 Gy, respectively [14]. In this regard, accumulating evidences exist on a reduced non-homologous end joining (NHEJ) repair response associated with HRS and persistent RAD51 foci, an essential component of the homologous recombination (HR) pathway at late time points after low-dose exposure [15]. This may indicate that a deregulation of both repair pathways may contribute to the non-linear induction of DSBs. Moreover, future investigations will further address a putative involvement of accumulation of DSBs at stalled replication forks [43] to contribute to the detection of residual γH2AX following a low-dose exposure especially in S-phase cells.

## Competing interests

The authors report no declaration of interest.

## Authors’ contributions

ML, SR and SH performed IF, ROS and SOD analyses and prepared the manuscript. CF participated in the design of the study and revised the manuscript critically. CR and FR supervised the analysis, and contributed substantially in preparing the manuscript. All authors read and approved the final manuscript.

## Supplementary Material

Additional file 1: Figure S1 Dose and time kinetics of γH2AX foci detection in EA.hy926 EC following low-dose X-irradiation differentiated in G1- and S/G2-phase cells. At 4 h before irradiation EA.hy926 EC were stimulated with TNF-α (20 ng/ml), while mock-treated cells served as a control. At 1 h, 4 h and 24 h post irradiation, cells were fixed, stained for γH2AX and CENP-F to differentiate G1 and S/G2 cell cycle phases. Data represent means ± SD from three independent experiments and a total of 40 G1- (A-C) and 40 S/G2-nuclei (D-F) per experiment. *p < 0.05 vs. 0.3 Gy and 0.7 Gy irradiated ECs. Click here for file

Additional file 2: Figure S2 SOD activity and SOD1 protein expression in primary HUVEC following low-dose X-irradiation. (A) Relative SOD activity as analysed at 24 h after irradiation by using a colorimetric activity assay. Data represent means ± SD (n = 3). *p < 0.05 vs. 0.3 Gy and 0.7 Gy treated cells. (B) Relative SOD1 protein expression at 24 h after irradiation normalized to β-actin control as determined by densitometric analysis of Western immunoblots (n = 2) using the ImageJ software package. *p < 0.05 vs. 0.3 Gy and 0.7 Gy treated cells. Click here for file
