# Supplementary material for: A non-linear detection of phospho-histone H2AX in EA.hy926 endothelial cells following low-dose X-irradiation is modulated by reactive oxygen species
Source: Radiat Oncol. 2014 Mar 22;9:80. doi: 10.1186/1748-717X-9-80 (PMC3997971; doi:10.1186/1748-717X-9-80)
Supplement: Additional file 1: Figure S1 — Dose and time kinetics of γH2AX foci detection in EA.hy926 EC following low-dose X-irradiation differentiated in G1- and S/G2-phase cells. At 4 h before irradiation EA.hy926 EC were stimulated with TNF-α (20 ng/ml), while mock-treated cells served as a control. At 1 h, 4 h and 24 h post irradiation, cells were fixed, stained for γH2AX and CENP-F to differentiate G1 and S/G2 cell cycle phases. Data represent means ± SD from three independent experiments and a total of 40 G1- (A-C) and 40 S/G2-nuclei (D-F) per experiment. *p < 0.05 vs. 0.3 Gy and 0.7 Gy irradiated ECs. [file 1748-717X-9-80-S1.pptx]

## Slide 1
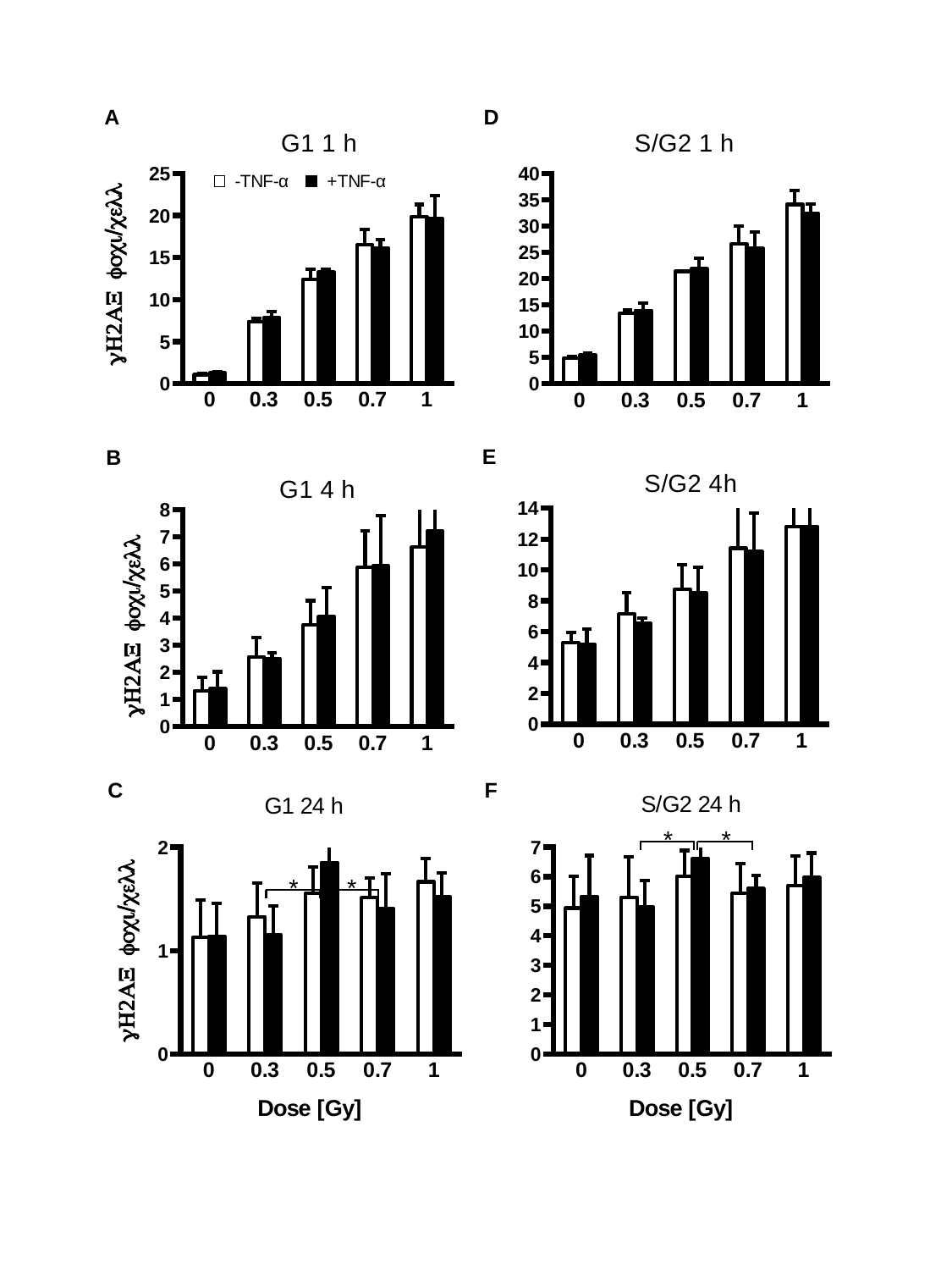

### Chart: G1 1 h
| Category | -TNF-α | +TNF-α |
|---|---|---|
| 0 | 1.075 | 1.275 |
| 0.3 | 7.399999999999999 | 7.841666666666666 |
| 0.5 | 12.41666666666667 | 13.25833333333333 |
| 0.7 | 16.5 | 16.16666666666667 |
| 1 | 19.8583333333333 | 19.625 |
### Chart: S/G2 1 h
| Category | -TNF-α | +TNF-α |
|---|---|---|
| 0 | 4.816666666666666 | 5.424999999999997 |
| 0.3 | 13.45 | 13.93333333333333 |
| 0.5 | 21.36666666666666 | 21.8583333333333 |
| 0.7 | 26.5583333333333 | 25.76004273504272 |
| 1 | 34.1 | 32.33333333333334 |A
D
### Chart: S/G2 4h
| Category | -TNF-α | +TNF-α |
|---|---|---|
| 0 | 5.275 | 5.158333333333333 |
| 0.3 | 7.141666666666667 | 6.566666666666666 |
| 0.5 | 8.738888888888885 | 8.525 |
| 0.7 | 11.40833333333333 | 11.19166666666667 |
| 1 | 12.78333333333333 | 12.80833333333333 |
### Chart: G1 4 h
| Category | -TNF-α | +TNF-α |
|---|---|---|
| 0 | 1.316666666666667 | 1.391666666666667 |
| 0.3 | 2.575 | 2.508333333333334 |
| 0.5 | 3.739141414141414 | 4.058333333333336 |
| 0.7 | 5.866666666666667 | 5.916666666666667 |
| 1 | 6.608333333333333 | 7.2 |E
B
### Chart: G1 24 h
| Category | -TNF-α | +TNF-α |
|---|---|---|
| 0 | 1.12857142857143 | 1.139285714285714 |
| 0.3 | 1.325 | 1.153571428571428 |
| 0.5 | 1.55357142857143 | 1.85 |
| 0.7 | 1.5125 | 1.404166666666667 |
| 1 | 1.664285714285715 | 1.52 |
### Chart: S/G2 24 h
| Category | -TNF-α | +TNF-α |
|---|---|---|
| 0 | 4.939285714285715 | 5.31785714285714 |
| 0.3 | 5.303571428571428 | 4.982142857142857 |
| 0.5 | 6.02142857142857 | 6.621428571428569 |
| 0.7 | 5.433333333333338 | 5.608333333333332 |
| 1 | 5.707142857142855 | 5.97 |C
F
*
*
*
*
