# Supplementary figures and images for: A non-linear detection of phospho-histone H2AX in EA.hy926 endothelial cells following low-dose X-irradiation is modulated by reactive oxygen species
Source: Radiat Oncol. 2014 Mar 22;9:80. doi: 10.1186/1748-717X-9-80 (PMC3997971; doi:10.1186/1748-717X-9-80)

## Slide 1
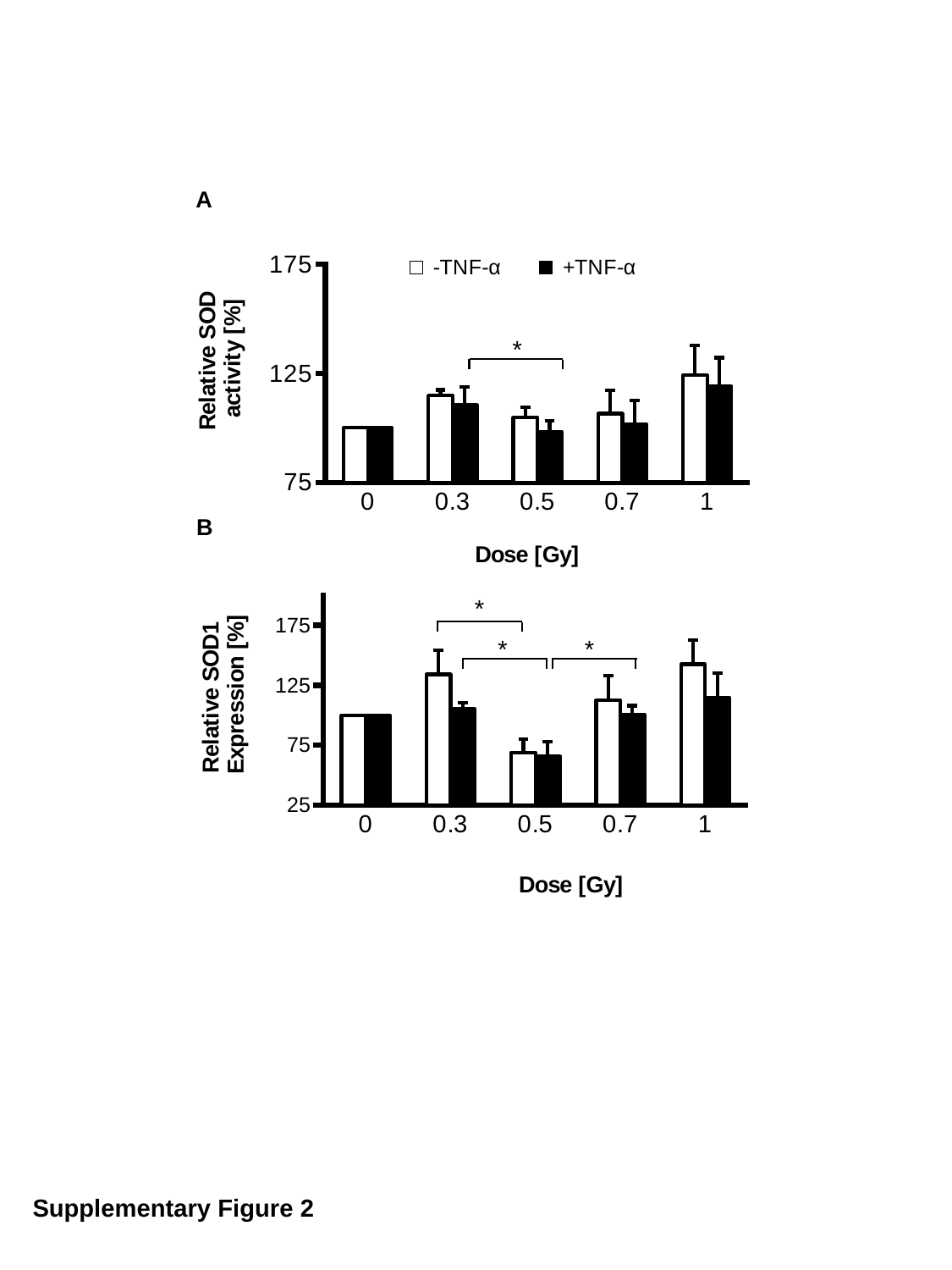

### Chart
| Category | -TNF-α | +TNF-α |
|---|---|---|
| 0 | 100.0 | 100.0 |
| 0.3 | 114.8148148148148 | 110.3508771929824 |
| 0.5 | 104.6296296296296 | 98.24561403508775 |
| 0.7 | 106.4814814814815 | 101.7543859649123 |
| 1 | 124.0740740740741 | 119.1666666666667 |A
*
### Chart
| Category | -TNF-α | +TNF-α |
|---|---|---|
| 0 | 100.0 | 100.0 |
| 0.3 | 134.0154352873464 | 105.0993319662581 |
| 0.5 | 69.0328182521564 | 66.2067009607753 |
| 0.7 | 112.3565527764576 | 100.624277590493 |
| 1 | 142.591419814847 | 114.8028639009254 |B
*
*
*
Supplementary Figure 2

Supplement: Additional file 2: Figure S2 — SOD activity and SOD1 protein expression in primary HUVEC following low-dose X-irradiation. (A) Relative SOD activity as analysed at 24 h after irradiation by using a colorimetric activity assay. Data represent means ± SD (n = 3). *p < 0.05 vs. 0.3 Gy and 0.7 Gy treated cells. (B) Relative SOD1 protein expression at 24 h after irradiation normalized to β-actin control as determined by densitometric analysis of Western immunoblots (n = 2) using the ImageJ software package. *p < 0.05 vs. 0.3 Gy and 0.7 Gy treated cells. [file 1748-717X-9-80-S2.pptx]
